# Strength

**Published:** 1875-05

**Authors:** 


### STRENGTH.

After all, there is nothing like strength. To secure it, is worth almost any effort; to retain it, will amply compensate the most ceaseless vigilance. Health may exist to a considerable extent, while strength is only partially developed; but in a symmetrical and well balanced organism, health and strength must go hand in hand.

There are various popular exercises employed to secure and retain strength, and all are worthy of consideration. Years ago, Dr. Windship, of Boston, astonished the country with his wonderful strength. He was able to lift an enormous weight, something like 1,600 lbs., we believe. This he did with the hands; with straps over the shoulders, he suspended about a ton and a quarter, if we remember right. Yet he was by no means a large or stalwart man. He was not a man of strong hereditary constitution. The whole secret of his great power was *gradual accumulation.* He commenced low, practiced regularly, and went to a marvelous figure. Out of Windship grew the "Health Lift," light-gymnastics, and the whole system of "cumulative exercise." A "reactionary" device was employed, giving a double pull, so that the act of carefully depositing a burden should be as useful, as a strengthening exercise, as the act of elevating it. All these appliances and methods have proved useful, and all have been rendered attractive by the pleasant, social atmosphere surrounding their use.

It is doubtful if any valuable purpose is subserved by the education of the muscles up to the accomplishment of enormous tasks. The great thing to be secured is such a reasonable exercise of *all* the muscles as shall secure for all, as well as for the blood vessels and nerves supplying and attending them, vigor and power. This can be accomplished, not by the mere act of lifting, although this act calls nearly all important muscles into play; but only by a variety of motions, in a variety of bodily attitudes. A man might be able to lift a ton, under favorable circumstances, and still be unable to elevate a bucket of water two feet from the ground, with his hand extended behind him to the full length of the arm. His failure would result from the fact that some new muscles would be summoned to the new duty, and that such of those employed as had been educated, had not been educated to lift in that precise way.

The idea which we wish to convey is simply this: that to give strength to all the muscles, all must be used, and also that all must be used in every position possible or likely to occur in ordinary life or in the event of accidents.

A device lately patented, and known as Goodyear's Exercising Tube, seems to accomplish the object. It is merely a series of strong, elastic tubes, made of "vulcanine," each provided with plugs at the end to serve as handles. A strong safety check or cord, the length of the tube when distended, is drawn through it and knotted outside the plugs. These tubes are to be pulled in many ways, either by holding an end in either hand, or by inserting one end in a post, or in a slotted board or stirrup, which may be held to the floor by the foot. In this way a simple lifting machine is secured, having every advantage which can be claimed by any of the costly devices in use, and involving only a trifling expense.

Many a weary brain-worker, who passes sleepless hours, when slumber should be upon him, would find relief by ten minutes exercise on this Tube. The blood—the tendency of which to the brain induces wakefulness—would be sent coursing to the extremities, and slumber would be pretty sure to follow.

But there is another use to which these little tubes can be put. It is all very well to talk about exercise and its advantages, but there are thousands of invalids and convalescents who are unable to lift themselves, to say nothing of lifting some hundreds of pounds. These will here find exercise within the limit of their feeble powers. The bed-ridden can secure exercise for a number of the muscles by having these tubes attached to the posts of the bedstead, to be toyed with and enjoyed as a salutary amusement, not less than as a strength-inducing recreation.

The inventor of these Exercising Tubes, Mr. Geo. W. Wood, No. 697 Broadway, New York, has published a circular setting forth the advantages of his system, and upon one side of the circular has given a series of graceful pictures exhibiting some of the modes of using the appliance. This circular he sends to all who apply. We have tested the Tubes, and have witnessed their use by others, and can earnestly recommend them as very valuable adjuncts to physical culture, and as occupying a place heretofore practically vacant. There is an exhileration attending their use which seems to accompany no other system of exercise, and this renders them very attractive to girls and boys, who return to the exercises with infinite zest. The attention of parents may well be given to the excellent means here provided for securing health and strength for those in their charge, not less than for themselves.

---

Electricity is worth more, in many forms of disease, than all the drugs known to science.

